# Impact of deep phenotyping: high diagnostic yield in a diverse pediatric population of 172 patients through clinical whole-genome sequencing at a single center

**DOI:** 10.3389/fgene.2024.1347474

**Published:** 2024-03-15

**Authors:** Ozlem Akgun-Dogan, Ecenur Tuc Bengur, Beril Ay, Gulsah Sebnem Ozkose, Emre Kar, Fuat Baris Bengur, Aybike S. Bulut, Ayca Yigit, Eylul Aydin, Fatma Nisa Esen, Ozkan Ozdemir, Ahmet Yesilyurt, Yasemin Alanay

**Affiliations:** ^1^ Division of Pediatric Genetics, Department of Pediatrics, School of Medicine, Acibadem University, Istanbul, Türkiye; ^2^ Rare Diseases and Orphan Drugs Application and Research Center (ACURARE), Acibadem University, Istanbul, Türkiye; ^3^ Department of Genome Studies, Health Sciences Institute, Acibadem University, Istanbul, Türkiye; ^4^ Division of Genetics and Genomic Medicine, Department of Pediatrics, Washington University School of Medicine, St. Louis, MO, United States; ^5^ School of Medicine, Acibadem University, Istanbul, Türkiye; ^6^ Acibadem Labgen Genetic Diagnosis Center, Istanbul, Türkiye; ^7^ Division of Medical Biology, Department of Basic Sciences, School of Medicine, Acibadem University, Istanbul, Türkiye

**Keywords:** whole-genome sequencing, first tier, diagnostic yield, undiagnosed patients, pediatric geneticist

## Abstract

**Background:** Pediatric patients with undiagnosed conditions, particularly those suspected of having Mendelian genetic disorders, pose a significant challenge in healthcare. This study investigates the diagnostic yield of whole-genome sequencing (WGS) in a pediatric cohort with diverse phenotypes, particularly focusing on the role of clinical expertise in interpreting WGS results.

**Methods:** A retrospective cohort study was conducted at Acibadem University’s Maslak Hospital in Istanbul, Turkey, involving pediatric patients (0–18 years) who underwent diagnostic WGS testing. Clinical assessments, family histories, and previous laboratory and imaging studies were analyzed. Variants were classified and interpreted in conjunction with clinical findings.

**Results:** The cohort comprised 172 pediatric patients, aged 0–5 years (62.8%). International patients (28.5%) were from 20 different countries. WGS was used as a first-tier approach in 61.6% of patients. The diagnostic yield of WGS reached 61.0%, enhanced by reclassification of variants of uncertain significance (VUS) through reverse phenotyping by an experienced clinical geneticist. Consanguinity was 18.6% of the overall cohort. Dual diagnoses were carried out for 8.5% of solved patients.

**Discussion:** Our study particularly advocates for the selection of WGS as a first-tier testing approach in infants and children with rare diseases, who were under 5 years of age, thereby potentially shortening the duration of the diagnostic odyssey. The results also emphasize the critical role of a single clinical geneticist’s expertise in deep phenotyping and reverse phenotyping, which contributed significantly to the high diagnostic yield.

## 1 Introduction

Children with undiagnosed conditions present a unique challenge in pediatric healthcare. They are often defined by at least one chronic condition that significantly impairs normal functioning, leading to a heavy reliance on a broad spectrum of healthcare services and specialists (Cohen et al., 2011; Kuo et al., 2016). Among the myriad of potential causes, Mendelian genetic disorders stand out as the most common underlying cause (Oei et al., 2017). Genetic evaluation of these patients is predominantly directed toward uncovering the origins of congenital anomalies and neurodevelopmental disorders (NDDs), encompassing conditions like developmental delay, intellectual disability, and autism spectrum disorder (ASD).

A comprehensive genetic assessment involves a detailed physical examination, an exploration of family history, and an analysis of previous laboratory findings and imaging studies. These insights guide the clinician in selecting the most suitable genetic test from a range of available methodologies designed to identify Mendelian conditions. The confirmation of a specific genetic diagnosis often concludes a diagnostic odyssey, heralding a new phase of individualized healthcare. Such a personalized medical strategy may encompass a series of additional diagnostic evaluations, directed referrals to specialized healthcare providers, continuous surveillance tailored to the specific condition, a range of therapeutic interventions, and genetic counseling for the patient’s family. Collectively, these measures offer a ray of hope to individuals and families who have tirelessly pursued a definitive explanation for their medical concerns (Boycott et al., 2019).

The era of genetic diagnosis for Mendelian conditions has evolved alongside the advancement of next-generation sequencing techniques, including whole-exome sequencing (WES) and whole-genome sequencing (WGS) (Stavropoulos et al., 2016; Lionel et al., 2018). WGS offers a thorough diagnostic capability by sequencing the entire genome, capturing a broad spectrum of causative variants, including coding single-nucleotide variants (SNVs), intronic and non-coding regulatory variants, copy number variants (CNVs), and mitochondrial genomic variants. The current guidelines from the American College of Medical Genetics (ACMG) suggest the use of WES and WGS as primary or secondary tests for individuals with congenital anomalies, developmental delay (DD), and intellectual disability (ID) (Manickam et al., 2021). Clinical interpretation of WES and WGS is vital to align molecular test findings with clinical features for a conclusive diagnosis. However, the diagnostic process may be complicated by varying interpretations and the identification of variants of uncertain significance (VUS), which are particularly challenging in cases especially with phenotypic diversity.

In this retrospective cohort study, conducted within a single center and led by a single clinical geneticist, we investigate the diagnostic yield of WGS while delving into the pivotal role of clinical expertise in the interpretation of WGS results. Furthermore, we elucidate the efficacy of employing a first-tier WGS approach in diagnosing a diverse spectrum of phenotypes among a cohort comprising 172 children with undiagnosed conditions, representing 21 different nationalities.

## 2 Materials and methods

### 2.1 Study overview

The study was designed as a retrospective cohort study. Undiagnosed pediatric patients (0–18 years) who underwent comprehensive genetic evaluations including diagnostic WGS at the Pediatric Genetics Unit of Acibadem University’s Maslak Hospital in Istanbul, Turkey, from January 2017 to August 2023 were included. Patient charts and diagnostic WGS results were retrospectively reviewed. This study was approved by the local Ethics Committee of Acibadem University (ATADEK-2019/14).

### 2.2 Cohort

Demographic information for 172 pediatric patients (aged 0–18 years) was retrieved from medical records. The data included the patients’ country of origin, gender, age, the age at the onset of initial symptoms, age at the time of testing, sibling history of a similar phenotype, consanguinity, dysmorphic features, and any prior genetic testing. Clinical findings were identified during pre-test evaluations by a single pediatric geneticist (YA, senior author). Human Phenotype Ontology (HPO) terms were employed to standardize the description of phenotypic abnormalities in patients. These terms were obtained from the Human Phenotype Ontology project, a widely recognized and continuously updated resource for human phenotypes, available at https://hpo.jax.org/app/. By recognizing that the HPO is still evolving and not all phenotypes have corresponding HPO terms, we carefully matched patient phenotypes with the most relevant available HPO terms. In instances where an exact HPO term was unavailable, we chose the closest matching term that could accurately depict the observed phenotype. Based on the prominent findings, we grouped patients into primary phenotype groups. These phenotype groups were as follows: NDDs (which includes ASD and non-ASD phenotypes such as neuromuscular disorders, epilepsy, and intellectual disability); musculoskeletal disorders (growth abnormalities, skeletal dysplasia, and connective tissue disorders); inherited metabolic disorders; and vascular, cutaneous, craniofacial, gastrointestinal, and genitourinary disorders.

### 2.3 Genomic analysis

WGS for diagnostic purposes was performed by CENTOGENE, a reference diagnostic laboratory based in Germany. Details regarding the genome sequencing methodology, bioinformatics pipeline, and reporting protocols are provided elsewhere (Bertoli-Avella et al., 2021). A single clinical geneticist evaluated each patient suspected of having a genetic disorder and delivered both pre- and post-test genetic counseling. Prior to testing, families were asked if they wished to be informed about incidental findings related to genes listed in the ACMG guidelines (Miller et al., 2023). Informed consent was obtained from all families for the diagnostic procedures.

### 2.4 Defining a diagnosis

Diagnostic results were categorized into three main categories as outlined elsewhere (Wright et al., 2018). These categories were determined by a combined approach: ACMG classification of the variants and reverse phenotyping by the clinical geneticist. The concept of “reverse phenotyping” in our study involves a comprehensive, post-test evaluation of the patient, conducted face-to-face. This process begins with a detailed explanation of the test results to the patient’s family. During the post-test visit, our focus intensified on the phenotype associated with the VUS . This includes a thorough physical examination, an in-depth review of the family history, and the implementation of a broad spectrum of diagnostic procedures. These procedures may encompass segregation analysis in family members, imaging studies to identify any structural anomalies, blood tests aimed at detecting metabolic or biochemical abnormalities, and neurological assessments to evaluate cognitive or motor functions. The purpose of these diverse diagnostic approaches is to either corroborate or refute the findings related to the VUS.

#### 2.4.1 Robust genetic diagnosis

This category includes patients in whom a pathogenic (P) or likely pathogenic (LP) variant (as per ACMG class I and II) was detected correlating directly with the patient’s phenotype.

#### 2.4.2 Likely genetic diagnosis

This category includes patients in whom a VUS variant (as per ACMG class III) was detected showing a potential association with the patient’s phenotype. Upon reporting a VUS (ACMG class III), a subsequent post-test clinical re-evaluation was performed by the clinical geneticist. The variant was deemed a “clinically relevant VUS,” and the patient was categorized under “likely genetic diagnosis” if the molecular test result was considered a likely cause after reverse phenotyping by the clinician.

#### 2.4.3 No genetic diagnosis

This group includes patients without a definitive causative variant or a variant of uncertain significance (ACMG class III) with no clear clinical relevance after reverse phenotyping by the clinical geneticist.

In the context of this paper, the term “solved” encompasses a “robust genetic diagnosis” and “likely genetic diagnosis” and cases who were subsequently diagnosed upon reanalysis. The remaining cases were categorized as “unsolved.” Reanalysis was performed by UDP-IST (www.istudp.istisna.org) and the diagnostic laboratory. All the variants were reviewed and reclassified according to the ACMG guidelines as of October 2023.

## 3 Results

### 3.1 Cohort characteristics

A single clinical geneticist conducted a comprehensive assessment of 172 pediatric patients aged 0–18 years between January 2017 and August 2023, each presenting with an undiagnosed condition suspected of genetic etiology. Within this cohort, 44.2% were female (*n*: 76) and 55.8% were male patients (*n*: 96), with an average age of 5.22 years and a median age of 4.53 years (range 0–18 years). Approximately one-third (34.3%) of the patients were under age 2 (Table 1)

**TABLE 1:** Demographic and phenotypic characteristics of the cohort.

Gender		*n*	Percentage (%)
	Female	76	44.2
	Male	96	55.8
Age at first referral			
	0–2	59	34.3
	≥2–5	49	28.5
	≥5–12	49	28.5
	≥12–18	15	8.7
Nationality			
	Turkish (four families live in EU)	123	71.5
	International (20 countries)	49	28.5
Consanguinity			
	Reported		
	Turkish	20/123	16.2
	International	12/49	24.4
	Denied	140	81.3
Primary phenotype			
	NDD	131	76.1
	ASD	16	9.3
	Non-ASD	115	66.8
	Others	41	23.8
	Musculoskeletal	18	
	Cutaneous	7	
	Metabolic	4	
	Gastrointestinal	3	
	Genitourinary	3	
	Craniofacial	5	
	Vascular	1	
Previous genetic testing			
	No	106	61.6
	Yes	66	38.3
	WES only	13	
	WES + CMA	4	
	CMA only	20	
	Karyotype	16	
	Other (NGS panels; Sanger seq.)	38	
WGS test design			
	Solo	148	86.0
	Trio	24	14.0

(EU, European Union; NDD, neurodevelopmental delay; ASD, autism spectrum disorder; WES, whole-exome sequencing; CMA, chromosomal microarray; seq., sequencing).

Patients of Turkish nationality constituted 71.5% (123/172) of the cohort, while the remaining 28.5% (49/172) were international, coming from 20 different countries (Supplementary Table S1). Consanguinity was reported in 32 families (18.6%). Consanguinity among Turkish families and international families was 16.1% and 24.4%, respectively. WGS was the first-tier diagnostic test for 61.6% of the patients, and of these, the age of 58.4% of them was less than 5 years.

### 3.2 Diagnostic yield

**FIGURE 1:**
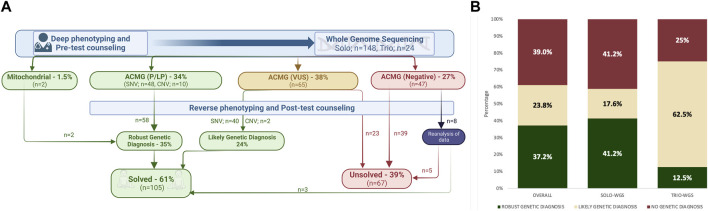
**(A)** Illustration summarizing the diagnostic process of the cohort; **(B)** diagnostic yield of whole-genome sequencing (WGS), comparing the specific rates for both solo and trio WGS within the cohort.

Figure 1A demonstrates and encapsulates the diagnostic process of the cohort. In the cohort, 61.0% (105/172) of the patients were either given a “robust genetic diagnosis” or classified with a “likely genetic diagnosis” and categorized as “solved.” The clinical geneticist engaged with each family on an average of four occasions. The initial consultations and pre-test counseling were held in person. The subsequent post-test counseling and follow-up assessments were performed face-to-face or via online platforms for international patients. The sessions typically lasted approximately 1 hour, and each was followed by providing the family and referring physicians with a detailed clinical note.

Molecular genetic testing findings in the “solved” group comprised SNVs, CNVs, and variations within the mitochondrial genome. In the study cohort (*n*: 172), WGS established a “robust genetic diagnosis” in 34.8% (60/172). VUS were found in 37.7% (65/172), and upon further assessment by the clinical geneticist, nearly two-thirds (42/65) of these VUS results were deemed clinically relevant for the patients’ phenotypes, thus categorized as “likely genetic diagnosis.” Negative WGS results were initially reported in 27.3% (47/172) of the cohort. Eight patients subsequently underwent reanalysis, and three of them were categorized within the “solved group” (Figure 1A).

The test design included 86.0% (*n*: 148) solo WGS and 14.0% (*n*: 24) trio WGS, which included both parents. In the solo-WGS group, a robust genetic diagnosis was initially made in 41.2% of patients, and this diagnostic rate increased to 58.7% following further post-test evaluation. The trio-WGS group had a 12.5% initial rate of “robust genetic diagnosis,” which increased to 75.0% after post-test evaluation. Figure 1B illustrates the diagnostic yield of WGS.

**FIGURE 2:**
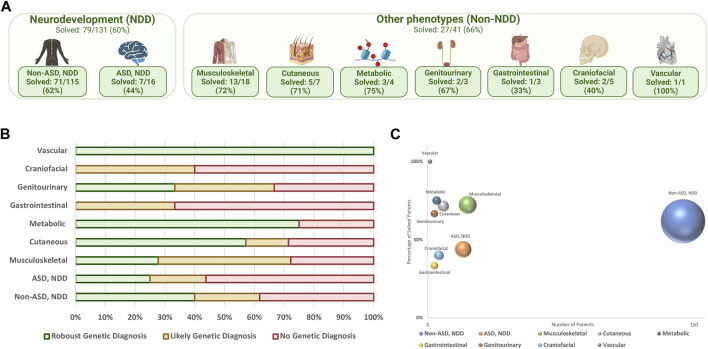
**(A)** Distribution of phenotype groups within the cohort, (%) solved patients; **(B)** final diagnostic result in different phenotype categories; **(C)** diagnostic yield of WGS in each phenotype category; the y-axis represents the percentage of solved patients, the x-axis shows the number of patients, and the size of the spheres correlate with sample size.

Figures 2A–C illustrate the diagnostic yield of WGS across different phenotype categories within the cohort. NDD was the most common phenotype, comprising 76.1% of the cohort, followed by musculoskeletal phenotypes. The average diagnostic yield within the NDD category was 59.5%, with higher rates in the non-ASD subgroup at 61.7%.

### 3.3 Findings in the solved cohort (*n*: 105)

**FIGURE 3:**
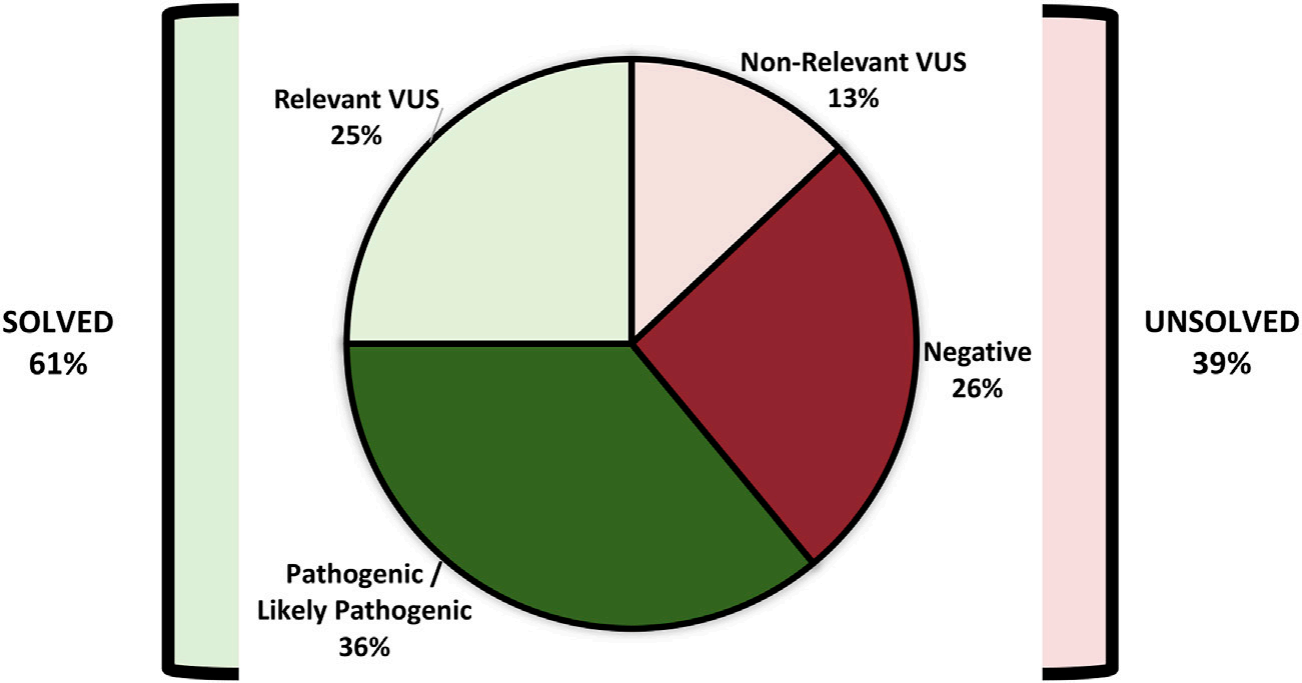
ACMG classification of variants in the study cohort (*n*: 172).

**FIGURE 4:**
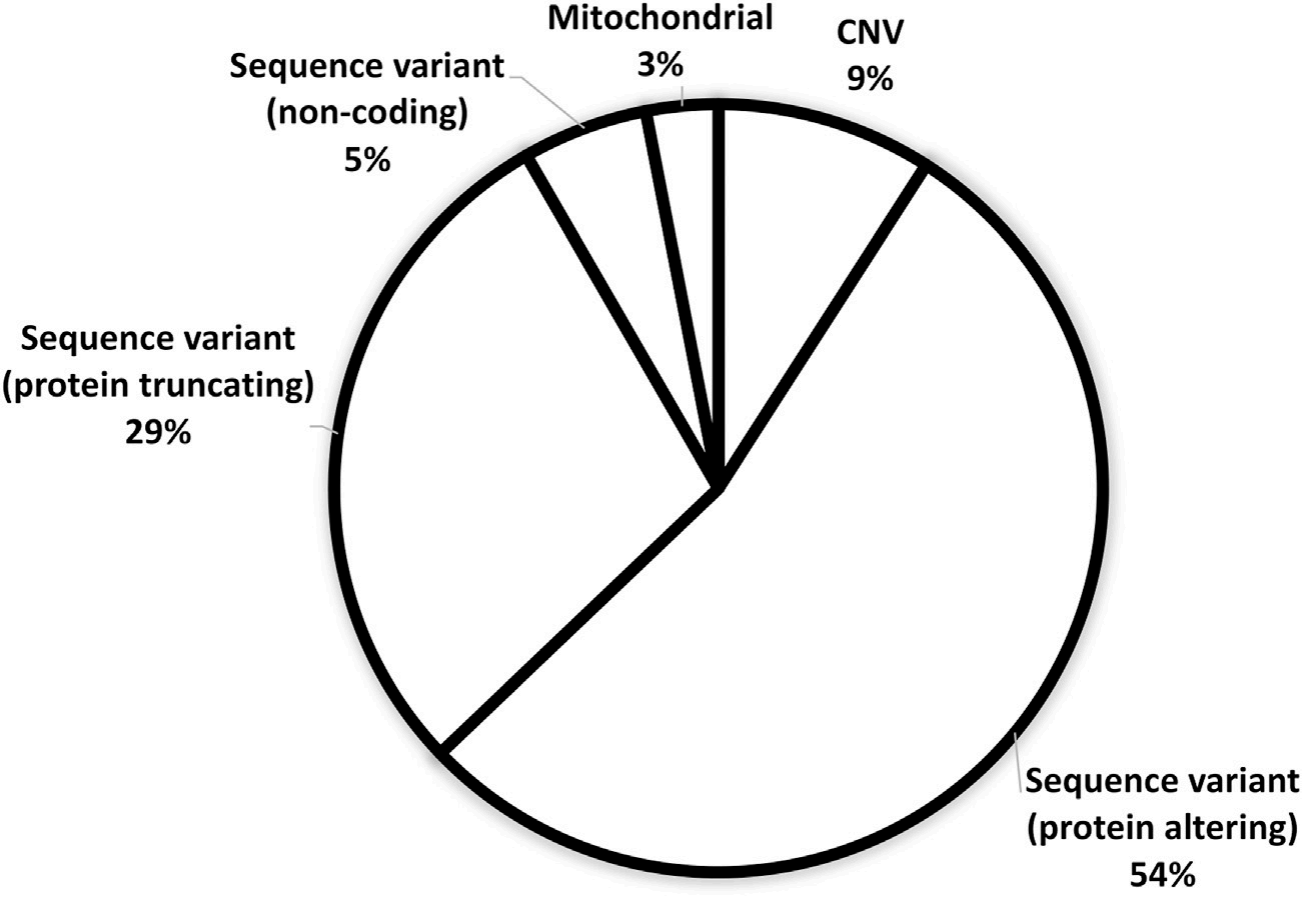
Distribution of variant types in the solved patient cohort (*n*: 105).

Figure 3 demonstrates the ACMG classification of variants in the study cohort (Supplementary Table S2). Figure 4 shows the distribution of the variant types in the solved cohort. Variants in nine solved patients have been previously reported (Bertoli-Avella et al., 2021). Notably, none of the patients with SVs had undergone chromosomal microarray (CMA) testing prior to this study. Mitochondrial variants were identified in two patients, where one had undergone both WES and CMA prior to WGS. Nine patients (8.5%) had a dual genetic diagnosis (Supplementary Table S3). On average, trio WGS reported 2.2 variants compared to the 1.13 variants of solo WGS. Among the 10 patients with prior negative WES reports, 50% were found to have variants in either non-coding regions or the mitochondrial genome.

Within this cohort, 45 patients were diagnosed with 47 monoallelic OMIM phenotypes. Among those with biallelic homozygous inheritance, 41.7% of parents denied consanguinity. The X-linked phenotype group comprised nine male patients and six female patients, including one male patient presenting with two X-linked phenotypes (Supplementary Table S2.). Dual diagnosis occurred in 8.5% of the solved cases, with one individual exhibiting two separate biallelic phenotypes. Table 2 shows the patterns of inheritance, prevalence of consanguinity, and the number of affected family members within the solved cohort.

**TABLE 2:** Solved patient group: inheritance pattern, consanguinity, and affected family members.

		Number of OMIM phenotypes	Reported consanguinity (%)	Affected parents/sibling
Monoallelic		47	3 (6.6%)	
	*De novo*	18		-
	Maternal	5		4
	Paternal	3		2
	Unknown	21		
Biallelic		38	16 (43.2%)	10
	Homozygous	25	14 (58.3%)	
	Compound heterozygous	13	2 (15.3%)	
X-linked		16	-	
	*De novo*	6		
	Maternal	7		1
	Unknown	3		
Mitochondrial		2	1 (50.0%)	

### 3.4 Selected solved patients

#### 3.4.1 Reanalysis with follow-up findings (P41)

Here, we present the case of a 6-month-old (corrected age-born 35 weeks) male infant, the first child of unrelated healthy parents (Supplementary Table S2). He had global developmental delay, hypertonicity in extremities, postnatal microcephaly, poor eye contact, and mild dysmorphic features. The EEG was normal, and brain MRI showed delayed myelination. Solo WGS reported a VUS variant in the *SIN3A* gene (MIM *607776); this variant was inherited from the mother and classified as non-relevant VUS. At the age of 2.5 years, he developed an abnormal EEG and was diagnosed with epilepsy. Brain MRI at 3 years of age showed cerebellar atrophy. He had severe global developmental delay. Reanalysis of solo WGS revealed heterozygous, *BRAT1* (MIM *614506), NM_152743.3:c.2324T>A, missense, VUS variant and heterozygous, *BRAT1*, NM_152743.3:c.1930C>T, nonsense, likely pathogenic variant. Parents were shown to be carriers.

#### 3.4.2 Dual diagnosis, dual inheritance, blended phenotype (P81)

Here, we present the case of a 19-month-old female infant, born at term as the first child of unrelated healthy parents. Her developmental milestones were delayed. She was nonverbal. Eye contact and eye pointing were present. She was aggressive, restless, and liked self-stimulation. Brain MRI revealed minimal dilatation of the third and lateral ventricles. She was diagnosed with hemolytic anemia at 7 months, requiring transfusions once every 5 weeks. The parental hematologic evaluations were normal. She had an open anterior fontanelle, prominent forehead, sparse eyebrows, broad nasal root, anteverted nares, and a depressed nasal tip. She had dysmorphic facial features, hyperhidrosis, very dry skin, curly and fine-wooly hair, and small hands and feet. Solo WGS revealed two novel variants, a heterozygous, *SPTB* (MIM *182870), NM_001355436.1:c.−52 + 1G>A, splice site, likely pathogenic variant and a heterozygous, *USP9X* (MIM *300072), NM_001039590.2:c.7237_7241del, frameshift, pathogenic variant. Segregation analysis showed that both variants were *de novo*. Reverse phenotyping by pediatric hematology confirmed the diagnosis of hereditary spherocytosis type 2 related to the *SPTB* variant, while the pathogenic variant in *USP9X* explained the ID/DD phenotype and most of the dysmorphic features. The family had a healthy second child.

#### 3.4.3 Missing CNVs in trans in a biallelic mitochondrial phenotype (P107)

Here, we present the case of a 5-month-old male infant, born at term to unrelated parents from the same district. He had no dysmorphic features. The medical history suggested severe anemia, gastrointestinal bleeding, and severe sensorineural hearing loss. Bone marrow examination showed dysplasia in all lineages. Laboratory results showed increased creatine kinase, increased lactate and pyruvate, and renal tubular acidosis. He had an undiagnosed sister, deceased at 18 months of age, with similar clinical findings through negative mitochondrial genome analysis. He had a previous negative WES result. Solo WGS revealed a heterozygous *COX10* (MIM *602125), NM_001303.3:c.635T>G, missense, VUS and a heterozygous 1.3-Mb deletion encompassing exon 7 of *COX10*. Segregation analysis of the parents confirmed the translocalization of the variants. The family later had a healthy child with preimplantation genetic diagnosis.

#### 3.4.4 Biallelic non-coding CNVs (P120)

Here, we present the case of a 7-year-old girl, with an average stature, born at term to first-cousin parents. She was born with bilateral club feet. Her birth measurements were average. The mesomelic shortness of the forearms and legs, Madelung’s deformity, ulnar deviation, and cubitus valgus were noted. Her height was at −1.45 SDS. X-rays showed mesomelic dysplasia. Solo WGS was analyzed with a focus on the *SHOX* (MIM *312865) gene region. Biallelic 199-Kb and 215-Kb duplication in the regulatory regions of the *SHOX* gene was determined as the cause of this mild Langer mesomelic dysplasia phenotype without short stature. The unaffected mother’s X-ray showed mild Madelung’s deformity. This is the first report of biallelic inheritance of both upstream and downstream duplicated CNVs of *SHOX*. The parents were counseled regarding autosomal recessive inheritance.

#### 3.4.5 Allelic heterogeneity and a novel single-exon deletion (P162)

Here, we present the case of a 2.5-year-old male infant, born at term as the first child of unrelated parents. He had average measurements. Slow linear growth was noted at 9 months of age. At 30 months, his height was 88.3 cm (−1.17 SDS). The father had proportionate short stature, cubitus valgus, and short 4–5th metacarpals. The father also had a paternal cousin with severe mesomelic short stature whose parents were first cousins. Physical examination revealed proportionate short stature, lordosis, relative macrocephaly, mild brachydactyly, and short toes with long halluces. Skeletal images did not show dysplasia. Solo WGS revealed a 22-Kb deletion, encompassing exons 13–22 of the *NPR2* (MIM *607072), resulting in a loss of one copy. Quantitative PCR analysis showed segregation from the father, suggesting monoallelic autosomal dominant inheritance. The father’s cousin’s photograph was shown; it was suggestive of a possible diagnosis of the biallelic acromelic dysplasia Maroteaux-type phenotype.

#### 3.4.6 Biallelic missing variant in trans (P168)

Here, we present the case of an 8-year-old female infant, born at term to unrelated healthy parents, who was referred for unexplained elevated liver enzymes detected incidentally. Liver biopsy findings were suggestive of glycogen storage disorders. Previous WES had revealed a heterozygous VUS in *GBE1* (MIM *607839). WGS showed two additional deep intronic VUS variants in *GBE1*. Segregation analysis revealed that one of the intronic VUS was in trans to the known VUS coding variant, supporting the diagnosis of glycogen storage disease type 1. Liver histology was an essential part of pre-test deep phenotyping.

## 4 Discussion

In 2021, the ACMG issued a recommendation supporting the use of WES/WGS as a first- or second-tier test for individuals with congenital anomalies, developmental delay, or intellectual disability (Manickam et al., 2021). The diagnostic yield of WGS was noted as 38% (Manickam et al., 2021). In 2023, the results from the DDD study in the UK and Ireland reported a 41% diagnostic yield (Wright et al., 2023). The authors discussed that the diagnostic yield represents a conservative estimate with higher yields anticipated if WGS had been offered as a first-tier investigation approach. The diagnostic yield in our cohort was observed to be 61.0%, notably exceeding that reported in the existing literature. This higher rate can be attributed to various factors, with the foremost being WGS as a first-tier test in 61.6% of the cohort (Table 2). The decision to prefer WGS as a first-tier test was made preceding the ACMG recommendations.

Based in Istanbul, our hospital is part of an international private healthcare system with many international offices. Istanbul is a hub where families from across Turkey and neighboring countries seek definitive diagnoses. The use of the first-tier WGS approach was higher among international patients (78.2%) than among Turkish citizens (53.8%) as CMA and clinical ES are covered by governmental health insurance in Turkey. Thirty percent of our cohort consists of international patients with limited time to stay for sequential testing. WGS was selected to conclude their diagnostic odyssey as swiftly as possible. Hence, the time interval from the onset of symptoms to definitive diagnosis is considerably shorter (mean 3.74 years). Another factor in choosing WGS as a first-tier test was the referral phenotype, namely, NDD in 72.6% of patients. First-tier WGS, providing analysis for CNVs, SNVs, and mtDNA, made it an optimal choice. The age profile of the cohort, with 34.3% of patients aged 0–2 years and 28.5% aged 2–5 years, also supports the relevance of using WGS as a first-tier test in early childhood, when timely intervention for rare genetic disorders is most pivotal.

In the solved patient cohort, it was found that 8% of the variants consist of CNVs, mitochondrial, and non-coding variants, which cannot be detected by WES analysis. This highlights the superior diagnostic efficacy of employing WGS as the primary testing approach, providing a notably greater contribution to the diagnostic rate than WES analysis. In the subset of patients with previous negative WES results, proceeding with WGS facilitated a diagnostic success rate of approximately 59% (10/17 cases). Of these 10 patients, five would likely have remained undiagnosed without WGS since the detected variants included mitochondrial variants (one patient), intragenic deletions (one patient), and intronic variants in trans with a pathogenic SNV (three patients). In the remaining five patients, coding SNVs were detected. These were overlooked in previous WES. This finding, along with those of previous studies, that demonstrate an increase of up to 56% in diagnostic yield by reanalyzing WES data in undiagnosed patients, highlights the critical need for reanalyzing existing WES data before proceeding to WGS (Jalkh et al., 2019). It also underscores the importance of conducting annual reassessments of data, particularly in patients with inconclusive WGS results.

The study cohort is also diverse in terms of consanguinity. Our Turkish cohort exhibited a consanguinity rate below both the national and Istanbul averages (Koc, 2022). Turkey ranks moderately on the global scale, with national averages between 20% and 25%. This rate increases as one moves eastward across different geographical regions. Istanbul, with its diverse population of 16 million reflects domestic migration from across Turkey, mirrors this national trend. The reason underlying lower consanguinity in our cohort may reflect the socioeconomic status of the cohort families. In Turkey, higher education and income levels are associated with reduced rates of consanguinity. WGS as an out-of-pocket test is more likely to be accessible to higher socioeconomic groups. Nonetheless, among the solved cases in our cohort, with homozygous biallelic variants, the consanguinity rate increased to 58%. This higher rate is particularly notable among families whose previous generations migrated from eastern and southeastern Turkey and international patients from northern Iraq and Saudi Arabia, where consanguinity rates are reported to exceed 50% (Monies et al., 2019). In the Turkish subgroup of the cohort, a significant majority (85.7%) of families who denied consanguinity, yet presented with homozygous biallelic variants, were indeed from the same geographical region. It is noteworthy that all international patients with biallelic homozygous variants, whose parents’ denied consanguinity, hailed from the Balkans and the Caucasus, underscoring the need for consideration of a common ancestry among families from these regions. These data collectively highlight the critical need for detailed assessment of individuals from homogeneous geographic regions for homozygous variants, regardless of self-reported consanguinity, given the substantial impact such factors have on the diagnostic process.

The rate of dual diagnoses within our cohort reached 8.5%, surpassing the range of 2.5%–7.2% documented in the existing literature (Rosina et al., 2022). On average, we identified 1.13 variants per singleton proband, in contrast to 2.2 variants per trio. In contrast to prior findings indicating a threefold increase in the incidence of multiple diagnoses within consanguineous families (Smith et al., 2019), our cohort, which featured a comparably diminished prevalence of consanguinity, exhibited a relatively modest occurrence, with merely three instances of dual diagnoses involving consanguinity and only one displaying double homozygous variants. This elevated rate of dual diagnoses, despite the lower-than-expected consanguinity prevalence, can be ascribed to the specialized nature of our center, which primarily serves as a referral hub for intricate or unconventional cases. Reports on multiple/dual diagnosis rates are mainly from WES studies; therefore, large diagnostic WGS cohorts may demonstrate a higher rate in the future.

To the best of our knowledge, this extensive single-clinician experience has not been previously documented in the literature. We believe that the substantial contribution of clinical expertise in deciphering next-generation sequencing (NGS) data plays a pivotal role in enhancing the diagnostic yield. Notably, in Turkey, the formal establishment of genetic counseling as a profession is yet to be realized, resulting in clinical geneticists assuming the responsibility of guiding patients through both pre- and post-test phases. This task, which can be particularly challenging, has been referred to as a “nightmare” in other countries (Eichinger et al., 2023).

In our cohort, the clinical geneticist engaged with each family on an average of four times. Initial consultations and pre-test counseling were conducted in person, ensuring a comprehensive understanding of the patient’s background. Subsequent post-test counseling and follow-up assessments took place either face-to-face or through online platforms. Each session typically lasted approximately 1 hour and was followed by the provision of detailed clinical notes to both the family and referring physicians. Despite the inherent difficulties, this methodology afforded us the opportunity to conduct meticulous deep phenotyping and reverse phenotyping, proving especially invaluable in the interpretation of variants of uncertain significance. The process of deep phenotyping, carried out by a single clinician with in-depth knowledge of each patient, involved providing the laboratory with more than 10 Human Phenotype Ontology terms per patient. This approach facilitated a comprehensive and tailored analysis, contributing significantly to our diagnostic capabilities. Furthermore, the same clinician’s continued follow-up of patients who initially received negative or VUS results, coupled with rigorous post-test reverse phenotyping and segregation analysis, positively impacted our overall diagnostic rate.

In conclusion, this study highlights how a single pediatric geneticist’s expertise, pre-test deep phenotyping, and post-test reverse phenotyping can significantly enhance the diagnostic yield of WGS. The findings particularly advocate for the selection of WGS as a first-tier testing in infants and children with rare diseases, who were under 5 years of age, thereby potentially shortening the duration of the diagnostic odyssey. Future studies are likely to reveal that first-tier WGS, when considering repeated visits and tests, may reduce the economic and psychosocial burden. This study cohort will continue to be evaluated over time with novel diagnostic tools, aiming to end the diagnostic odyssey for those remaining undiagnosed.

## Data Availability

The original contributions presented in the study are included in the article/Supplementary Material; further inquiries can be directed to the corresponding author.
